# Supplementary material for: Preserved cerebellar functions despite structural degeneration in older adults
Source: eLife. 2026 Sep 7;15:RP109440. doi: 10.7554/eLife.109440 (PMC13549622; doi:10.7554/eLife.109440)
Supplement: Peer review history

# Editor report: eLife Assessment

**DOI:** 10.7554/eLife.109440.3.sa0

**Authors:** Alaa A Ahmed

**Affiliations:** University of Colorado Boulder United States

**Keywords:** Compelling, Important

This **important** study examined age-related changes in cerebellar function by testing a large sample of younger and older adults, including 30 over 80 years old, on motor and cognitive tasks linked to the cerebellum and conducting structural imaging. Their findings show that cerebellar-dependent functions are mostly maintained or even enhanced across the lifespan, with cerebellar-mediated motor abilities remaining intact despite degeneration, in contrast to non-cerebellar measures. Overall, the authors provide **compelling** evidence in support of preserved cerebellar function with age. These results highlight the resilience and redundancy of cerebellar circuits and offer key insights into aging and motor behavior.

# Referee report: Reviewer #1 (Public review)

**DOI:** 10.7554/eLife.109440.3.sa1

Summary:

Witte et al. examined whether canonical behavioral functions attributed to the cerebellum decline with age. To test this, they recruited younger, old, and older-old adults in a comprehensive battery of tasks previously identified as cerebellar-dependent in the literature. Remarkably, they found that cerebellar function is largely preserved across the lifespan-and in some cases even enhanced. Structural imaging confirmed that their older adult cohort was representative in terms of both cerebellar gray- and white-matter volume. Overall, this is an important study with strong theoretical implications and compelling evidence supporting the motor reserve hypothesis, demonstrating that cerebellar-dependent measures remain largely intact with aging.

Strengths:

(1) Relatively large sample size.

(2) Most comprehensive behavioral battery to date assessing cerebellar-dependent behavior.

(3) Structural MRI confirmation of age-related decline in cerebellar gray and white matter, ensuring representativeness of the sample.

Weaknesses:

The absence of a voxel-based morphometry (VBM) analysis limits the anatomical and functional specificity of the conclusions. Such an analysis would help identify which functions are truly cerebellar-dependent, rather than relying primarily on inferences drawn from prior neuropsychological literature. Notably, the authors have undertaken this analysis in a separate manuscript.

As acknowledged in the Discussion, the classification of tasks as "cerebellar-dependent" versus "general" remains somewhat ambiguous. Some measures labeled as "general" may still engage cerebellar processes. Moreover, analyses in the authors' forthcoming manuscript show weak structure-behavior correlations, casting further doubt on how clearly cerebellar-specific functions can be distinguished from more general processes.

# Referee report: Reviewer #2 (Public review)

**DOI:** 10.7554/eLife.109440.3.sa2

Summary:

The authors are investigating cerebellar-mediated motor behaviors in a large sample of adults, including 30 individuals over the age of 80 (a great strength of this work). They employed a large battery of motor tasks that are tied to cerebellar function, in addition to a cognitive task and motor tasks that are more general. They also evaluated cerebellar structure. Across their behavioral metrics, they found that even with cerebellar degeneration, cerebellar-mediated motor behavior remained intact relative to young adults. However, this was not the case for measures not directly tied to cerebellar function. The authors suggest that these functions are preserved and speak to the resiliency and redundancy of function in the cerebellum. They also speculate that cerebellar circuits may be especially good for preserving function in the face of structural change. The tasks are described very well, and their implementation is also well-done with consideration for rigor in the data collection and processing. The inclusion of Bayesian estimates is also particularly useful, given the theoretically important lack of age differences reported. This work is methodologically rigorous with respect to the behavior, and certainly thought-provoking.

Strengths:

The methodological rigor, inclusion of Bayesian statistics, and the larger sample of individuals over the age of 80 in particular are all great strengths of this work. Further, as noted in the text, the fact that all participants completed the full testing battery is of great benefit. Please note, upon my second review the strengths remain. This is a really wonderful investigation and amazingly comprehensive from a behavioral perspective given the numerous tasks and domains that were considered.

Weaknesses:

The suggestion of cerebellar reserve, given that at the group level there is a lack of difference for cerebellar specific behavioral component,s could be more robustly tested. That is, the authors suggest that this is a reserve given that volume of cerebellar gray matter is smaller in the two older groups, though behavior is preserved. This implies volume and behavior are seemingly dissociated. However, there is seemingly a great deal of behavioral variability within each group and likewise with respect to cerebellar volume. Is poorer behavior associated with smaller volume? If so, this would suggest still that volume and behavior are linked; but, rather than being age that is critical it is volume. On the flip side, a lack of associations between behavior and volume would be quite compelling with respect to reserve. More generally, as explicated in the recommendations, there are analyses that could be conducted that, in my opinio,n would more robustly support their arguments given the data that they have available.

The authors have done wonderful work to address the comments from the initial feedback/reviews. While I may ultimately disagree with the approach of including the imaging data in another manuscript, that is at the same time, a reasonable decision. This, however, does not change the impression that the paper would be stronger with the inclusion of the volumetric imaging data. I can understand why it may be published separately - it would be a very long paper to include both. At the same time the assertions made here, which are largely nicely supported by the preprint, would ultimately strengthen this work. The behavior certainly stands on its own as an excellent and needed investigation; together, both pieces make for a truly excellent contribution to the literature.

# Author comment: Author response

**DOI:** 10.7554/eLife.109440.3.sa3

**Authors:** Anda de Witte, Anouck Matthijs, Benjamin Parrell, Dante Mantini, Jolien Gooijers, Jean-Jacques Orban de Xivry

**Affiliations:** KU Leuven Leuven Belgium; KU Leuven Leuven Belgium; University of Wisconsin-Madison Madison United States; KU Leuven Leuven Belgium; KU Leuven Leuven Belgium; KU Leuven Leuven Belgium

The following is the authors’ response to the original reviews.

**Public Reviews:**

**Reviewer #1 (Public review):**

Summary:

Witte et al. examined whether canonical behavioral functions attributed to the cerebellum decline with age. To test this, they recruited younger, old, and older-old adults in a comprehensive battery of tasks previously identified as cerebellar-dependent in the literature. Remarkably, they found that cerebellar function is largely preserved across the lifespan-and in some cases even enhanced. Structural imaging confirmed that their older adult cohort was representative in terms of both cerebellar gray- and white-matter volume. Overall, this is an important study with strong theoretical implications and convincing evidence supporting the motor reserve hypothesis, demonstrating that cerebellar-dependent measures remain largely intact with aging.

Strengths:

(1) Relatively large sample size.

(2) Most comprehensive behavioral battery to date assessing cerebellar-dependent behavior.

(3) Structural MRI confirmation of age-related decline in cerebellar gray and white matter, ensuring representativeness of the sample.

Weaknesses:

(1) Although the authors note this was outside the study's scope, the absence of a voxel-based morphometry (VBM) analysis limits anatomical and functional specificity. Such an analysis would clarify which functions are cerebellar-dependent rather than solely inferring this from prior neuropsychological literature.

(2) As acknowledged in the Discussion, task classification (cerebellar-dependent vs. general measures) remains somewhat ambiguous. Some "general" measures may still rely on cerebellar processes based on the paper's own criteria - for example, tasks in which individuals with cerebellar degeneration show impairments.

(3) Cerebellar-dependent and general measures may inherently differ in measurement noise, potentially biasing results toward detecting effects in general measures but not in cerebellar-dependent ones.

We appreciate Reviewer #1's positive assessment of the study, including the acknowledgment of our large sample size, comprehensive behavioral battery, and verification of cerebellar atrophy using MRI. We address the concerns raised as follows:

(1) Voxel-based morphometry (VBM) and anatomical specificity

We agree that VBM would strengthen anatomical specificity. As noted in our response to private comments, we have carried out these analyses as part of a separate dedicated study, now available as a preprint (“Aging is associated with uniform structural decline across cerebellar regions while preserving topological organization and showing no relation with sensorimotor function”, https://doi.org/10.64898/2026.02.13.705695). This work investigates region-level cerebellar aging and its relationship with behavior in detail, including both anatomical and functional parcellations. In short, the preprint demonstrates the absence of structure-function relationship between cerebellar regions (from either anatomical or functional atlases) and cerebellar function. Given the scope of the present manuscript, which focuses primarily on behavioral evidence for cerebellar preservation, we chose not to expand this paper further with VBM results.

(2) Task classification and cerebellar involvement

We clarified in the revised manuscript that even “general” measures likely involve cerebellar processing to some extent. We have strengthened the discussion explaining that these measures do not primarily depend on cerebellar function, in contrast to the cerebellar-specific metrics derived from established models (e.g., clock variance in rhythmic tapping). We now explicitly caution against interpreting these general measures as cerebellar-independent.

(3) Measurement noise and differential sensitivity

To address the reviewer’s concern that measurement noise may differ between task categories, we now report split-half reliabilities for all measures in the Supplement. These data demonstrate no systematic reliability disadvantage for cerebellar-specific tasks that could explain the pattern of results.

**Reviewer #2 (Public review):**

Summary:

The authors are investigating cerebellar-mediated motor behaviors in a large sample of adults, including 30 individuals over the age of 80 (a great strength of this work). They employed a large battery of motor tasks that are tied to cerebellar function, in addition to a cognitive task and motor tasks that are more general. They also evaluated cerebellar structure. Across their behavioral metrics, they found that even with cerebellar degeneration, cerebellar-mediated motor behavior remained intact relative to young adults. However, this was not the case for measures not directly tied to cerebellar function. The authors suggest that these functions are preserved and speak to the resiliency and redundancy of function in the cerebellum. They also speculate that cerebellar circuits may be especially good for preserving function in the face of structural change. The tasks are described very well, and their implementation is also well-done with consideration for rigor in the data collection and processing. The inclusion of Bayesian estimates is also particularly useful, given the theoretically important lack of age differences reported. This work is methodologically rigorous with respect to the behavior, and certainly thought-provoking.

Strengths:

The methodological rigor, inclusion of Bayesian statistics, and the larger sample of individuals over the age of 80 in particular are all great strengths of this work. Further, as noted in the text, the fact that all participants completed the full testing battery is of great benefit.

Weaknesses:

The suggestion of cerebellar reserve, given that at the group level there is a lack of difference for cerebellar-specific behavioral components, could be more robustly tested. That is, the authors suggest that this is a reserve given that the volume of cerebellar gray matter is smaller in the two older groups, though behavior is preserved. This implies volume and behavior are seemingly dissociated. However, there is seemingly a great deal of behavioral variability within each group and likewise with respect to cerebellar volume. Is poorer behavior associated with smaller volume? If so, this would still suggest that volume and behavior are linked, but rather than being age that is critical, it is volume. On the flip side, a lack of associations between behavior and volume would be quite compelling with respect to reserve. More generally, as explicated in the recommendations, there are analyses that could be conducted that, in my opinion, would more robustly support their arguments given the data that they have available. This is a well-executed and thought-provoking investigation, but there is also room for a bit more discussion.

We appreciate Reviewer’s recognition of the methodological rigor of the study. The public review focuses on the structure-function relationship for the cerebellum. Given that the volume of the cerebellum is smaller in older adults but that the identified cerebellar function are maintained, we conclude that there is no structure-function relationship. We agree with the reviewer that this could be tested further by looking at different parcellations of the cerebellum and demonstrating the absence of association between smaller regions of the cerebellum and the investigated cerebellar function. We agree with the reviewer that this is interesting but believe that this goes beyond the scope of this already extensive paper. For this reason, detailed analyses of the structure-function relationship are available in the preprint version of another paper entitled “Aging is associated with uniform structural decline across cerebellar regions while preserving topological organization and showing no relation with sensorimotor function”, (https://doi.org/10.64898/2026.02.13.705695). In this preprint, across multiple anatomical and functional parcellations, we found no meaningful association between cerebellar structure and cerebellar-specific behavioral measures.

**Recommendations for the authors:**

**Reviewer #1 (Recommendations for the authors):**

Prefacing these suggestions, I want to commend the authors for undertaking this Herculean effort, recruiting such a large sample and administering an extensive battery of tasks. This is an impressively comprehensive study!

(1) Lesion-symptom mapping. The authors state that lesion-symptom mapping was beyond the scope of the study, but it is unclear why such an analysis could not be performed. Including it would strengthen inferences linking cerebellar structure to behavioral outcomes and help differentiate cerebellar-specific from general performance measures.

(2) Inter-measure correlations. For cerebellar-dependent tasks, did the authors examine correlations among behavioral measures? If cerebellar aging effects are relatively uniform across the cerebellar cortex, performance across tasks engaging distinct cerebellar regions should, in theory, covary. Similar pairwise correlations for general measures could provide a useful comparison.

1 + 2: We fully agree with this two points; however, we decided to address this analysis in a separate paper. In the current manuscript, our primary focus was on the behavioral aspects, as these are already quite extensive on their own. In our subsequent work, we conducted an in-depth investigation into the relationship between cerebellar-specific measures and cerebellar structure across distinct cerebellar regions (including anatomical regions and functionally defined regions according to the atlas of Nettekoven et al., 2024). We found that aging does not affect the cerebellum uniformly, but that some anatomical regions exhibit stronger age effects. For the functionally defined regions the age effects were uniformly though. There was no relation between behavioral cerebellar-specific measures and regional gray matter structure.

In this second paper we also analyzed inter-measure correlations between behavioral cerebellar-specific measures. We did not find any correlations between cerebellar outcomes of different tasks, which indeed could indicate that the different tasks engage distinct cerebellar regions. In addition, we did not find any relation between cerebellar outcomes and anatomically or functionally defined cerebellar regions.

You can find a preprint of the second manuscript entitled “Aging is associated with uniform structural decline across cerebellar regions while preserving topological organization and showing no relation with sensorimotor function” here: https://doi.org/10.64898/2026.02.13.705695

(3) Measurement sensitivity. Could differences in age effects reflect varying measurement noise between cerebellar-specific and general measures? For instance, even among younger participants, cerebellar-related measures (e.g., slope in mental rotation) might exhibit greater variability - given that they depend on more conditions, each with its own noise - than general metrics (e.g., baseline motor variability or choice reaction time estimated from a single condition). This could affect sensitivity to detect age-related change and bias results toward finding effects in general rather than cerebellar-specific measures.

To address this concern, we computed split-half reliability for both cerebellar-specific and general sensorimotor measures and added these estimates to the supplementary materials. As can be seen from Author response table 1, there is no consistent pattern of lower reliability for cerebellar-specific measures that could plausibly account for the absence of age-related effects.

**Author response table 1.** Split-half reliabilities.

Task	Cerebellar- specific				General sensorimotor			
	Measure	Young	Older	Older-Old	Measure	Young	Older	Older-Old
Rhythmic finger tapping	Clock variance	0.66	0.79	0.89	Rhythm deviation	0.98	0.98	0.99
Grip force adjustment	Grip force anticipation	0.94	0.92	0.84	Grip safety margin	-	-	-
Inter-joint coordination	Shoulder anticipation	0.87	0.83	0.89	Shoulder deviation	0.98	0.99	0.98
Eye movement coordination	Eye velocity anticipation	0.55	0.61	-	Eye tracking accuracy	0.97	0.97	-
Force matching	Sensory attenuation	0.94	0.94	0.96	Sensory accuracy	0.73	0.84	0.86
Reach adaptation	Reach adaptation	0.96	0.98	0.98	Reach accuracy	0.41	0.43	0.60
Speech adaptation	Speech adaptation	0.99	0.99	0.98	Speech variance	0.90	0.79	084
Mental rotation	Mental rotation pace	0.73	0.86	0.71	Choice reaction time	0.91	0.92	0.94

(4) Task dependence on the cerebellum. It is difficult to argue that measures such as reach accuracy, choice reaction time, or rhythm deviation are non-cerebellar. Ataxia certainly impacts reach accuracy. Although patient evidence is mixed - and even when there is a lack of dissociation (e.g., prolonged choice reaction times in both cerebellar and PD groups) - this does not preclude cerebellar involvement in these measures. Indeed, as the authors stated, claims of cerebellar independence should therefore be made cautiously (can be addressed by VBM in comment 1).

In the paper we tried to emphasize that the general sensorimotor measures still involve cerebellar functions, as this is the case with many movement-related measures. However we theorized that they do not primarily depend on cerebellar function. For example rhythm deviation in the finger tapping task is influenced by cerebellar timing mechanisms as well as motor execution noise, attention, etc. While the cerebellar-specific measure from this task, which is the clock variance, has been shown to extract the contribution of cerebellar-dependent timing mechanisms to this task (Ivry & Keele, 1989).

On p.37, we added the following paragraph:

“Similarly, it is important to recognize that general sensorimotor performance is not independent of cerebellar processing. Many broad measures, such as movement accuracy, reaction time, likely reflect contributions from many different brain regions including the cerebellum. As a result, age‑related differences in general sensorimotor performance may emerge from multiple interacting systems rather than cerebellar function alone.”

(5) Interpreting preserved or enhanced function. The finding of preserved - or even enhanced - performance in older adults is compelling. The authors interpret this as evidence for cerebellar reserve or compensation for cortical decline. An alternative explanation is that cerebellar structures simply decline more slowly than cortical ones, as their gray-matter data suggest; so rather than cerebellar activity revving up, it may remain the same: For example, following up on several of the authors' prior papers, Cisneros et al. (2024) reported enhanced implicit recalibration with age, potentially reflecting greater reliance on cerebellar forward models as sensory (especially proprioceptive) signals degrade. However, this may reflect reweighting rather than compensation - where cerebellar contributions are not enhanced, but rather preserved as other systems decline more rapidly. It would be valuable for the authors to clarify whether they view their findings as evidence of reweighting (slower decline) or compensation (increased contribution).

We completely agree with this additional interpretation and added a small section to the discussion about it. However, based on the structural cerebellar measures that we have, it is difficult to state whether the reweighting or compensation theory would be more plausible. In either way, both are in line with the cerebellar reserve theory

Added to discussion (P. 35):

Importantly, the relative preservation of cerebellar structure compared to other systems may itself contribute to the maintained cerebellar function observed in older age. Even if structural decline is present, the fact that it progresses more slowly than in many cortical and subcortical regions suggests that a form of structural reserve remains available in the cerebellum. This structural reserve could underlie the continued efficiency of cerebellar circuits and support their capacity to sustain motor functions across aging.

(6) Mental rotation and the continuity hypothesis. The age-related decline in mental rotation performance, if cerebellar-dependent (see McDougle et al., 2022; note minor inconsistency in citation format throughout the paper), supports emerging theories that the cerebellum supports continuous mental simulations in both cognition and action, whether it's forward model simulation or interval-based timing in the motor control domain or mental rotation/intuitive physics in the cognitive domain (Tsay & Ivry, 2025). Given that mental rotation showed the strongest age effect, it would be fascinating to examine whether this correlates with structural loss in Crus I/II, regions most implicated in higher-order cognitive functions - related to Comment 1 above. Even on a crude level, without correlating with behaviour, do the authors have a map for which areas show greater degeneration than others?

This is also something we did in the other paper mentioned before (Figure 5 of the new preprint). At a first glimpse, the mental rotation outcomes show a strong positive correlation with Crus I and a negative correlation with Crus II, however none of these were significant and the fact that their sign is opposite suggest that these might be random. Indeed, in the preprint, we also compare age-related changes in grey matter volumes for different anatomical and functional cerebellar regions (Figure 1).

The inconsistencies in citation format have been fixed as well.

(7) Continuous age analyses. An exploratory analysis correlating age (as a continuous variable) with each dependent measure might provide greater sensitivity than categorical group comparisons, revealing more graded relationships between age and performance.

Our experiment was not designed to perform such analysis. Testing for group differences provides more power than testing for correlations. For this reason, given that our clearly separated age groups did not show any behavioral differences, we do not expect such an analysis to provide substantial additional insight. Given that the paper is already very extensive, we haven’t performed this additional analysis.

Congratulations on this comprehensive piece of work!

Thank you for your kind words

**Reviewer #2 (Recommendations for the authors):**

In the introduction, the authors note that the current literature on the cerebellum in aging has evidence from "studies that relied on single-task paradigms", including a citation to an eye-blink conditioning study. They then note "instead of capturing a broader range of specific cerebellar functions". What do they mean by this? Eye-blink conditioning, for example, when administered in a delay paradigm, is tied directly to the cerebellum and is arguably a cerebellar function or learning paradigm. Some clarity about his point is needed.

The meaning of this is that most previous studies examining cerebellar function in older adults relied on a single task, or on tasks that were functionally very similar, such as balance and gait, to assess performance. In contrast, our study incorporated multiple tasks targeting different sensorimotor skills, allowing us to identify broader patterns in cerebellar sensorimotor performance in older adults.

To make this clearer, we have rephrased the sentence (p.4):

“However, much of the evidence supporting this theory comes from studies that narrowly focused on a single task (Boisgontier & Nougier, 2013; Miller et al., 2013; Woodruff-Pak et al., 2001) or on assessments within similar cerebellar domains such as balance and gait (Droby et al., 2021; Rosano et al., 2007), instead of capturing a broader range of specific cerebellar functions.”

The authors note that many cerebellar tasks that are impaired in patients are preserved in older adults. The authors, however, seem to ignore delay eyeblink conditioning. Gerwig and colleagues (2010, Behav Brain Res) have shown that this is impacted in patients, and it is also robustly impacted in aging. Older adults still learn, but the age effects are highly replicable. A clear discussion of eye-blink conditioning and how it fits into this framework, and with your findings here, would be really helpful. It seems like a notable oversight not to have it discussed, given the age effects in this context, even if it was not included as a measure.

Eye blink conditioning is an interesting example that seems to contradict our theory: eye-blink conditioning is both affected by age and dependent on the cerebellum. However, while age-related changes in cerebellar structure evolve continuously with age, changes in eye-blink conditioning performance remains unchanged between 40 and 80 years old. Therefore, eye-blink conditioning suggest that age-related changes in cerebellar structure are not related to possible age-related changes in function. This discussion was already included in the manuscript on p. 36, which reads as:

“Similarly, no eye-blink conditioning task was included, as it is heavily influenced by cognitive factors such as awareness and arousal, and fear conditioning (LaBar et al., 2004). Previous work has shown that many variables, such as blink reaction time and motor components of the eyeblink reflex, introduce substantial variability in responses at older age (Woodruff-Pak & Jaeger, 1998). In contrast, this study found that only performance on the rhythmic finger-tapping task, similar to what we included in our battery, emerged as a significant predictor of age-related differences in eye-blink conditioning. Furthermore, age-related differences appeared to plateau after early adulthood, with no significant variation in the percentage of correct responses between ages 40 and 80 (Woodruff-Pak & Jaeger, 1998). Practically, the extended duration of the training protocol also makes this task unsuitable for inclusion in a test battery (Winton et al., 2025).”

This approach also does not consider variability within older adults. That is, on average, they may do better than patients. But, there are also individual differences in cerebellar metrics (structure, for example) within an older adult sample that are a critical consideration here. When looking at the behavioral plots that include the individual data points (which is a great addition and very helpful), it is clear that variability is prevalent. As noted below, it may still be that cerebellar metrics are associated with behavior, given the high degree of variability within the groups across aging.

We agree with the reviewer that variability is prevalent, as it is in any experiment. In our latest preprint entitled “Aging is associated with uniform structural decline across cerebellar regions while preserving topological organization and showing no relation with sensorimotor function” (https://doi.org/10.64898/2026.02.13.705695), we investigated whether variability in cerebellar structure could predict variability in cerebellar functions. Across all our tasks, we did not find such association, independently of whether we defined cerebellar regions based on an anatomical atlas or a functional one.

The use of 23 as the cut-off for MOCA scores is rather low. What was the justification for this within the literature? The authors note wanting to ensure task instructions and those with symptoms of potential MCI, but often 26 is used as a minimum score (with 25 and below being potential MCI).

In the methods, we refer to the study of Carson et al. (2018) that recommends a cutoff score of 23/30 instead of 26/30 as it shows overall better diagnostic accuracy. We selected this cutoff to emphasize that our sample was not restricted to only the highest‑performing older adults. However, we agree that this is not sufficiently explained in the text, so we briefly clarified this (p.5):

“We assessed cognitive functioning in both older and older‑old participants using the Montreal Cognitive Assessment (MoCA). A minimum score of 23 out of 30 was required for inclusion, following the recommendation by Carson et al. (2018), who demonstrated that this reduced cutoff yields fewer false positives and provides better overall diagnostic accuracy than the original 26/30 threshold. We adopted this criterion to ensure that our sample was not limited to only the highest‑performing older adults.”

The authors note that the timing of the visits was adapted based on participant availability. It would be helpful to report the mean length of time between sessions, as well as the range.

We added this to the method section (p.6):

“There was no fixed interval between the two behavioral sessions. Ideally, both were scheduled within one week, but in practice, the timing was adapted to participants’ availability. Across all participants, this resulted in a mean inter-session interval of 7.40 days (± 9.03; range = 0-63 days). The average interval between the behavioral sessions and the MRI scanning was 6.86 days (± 8.90; range = 0-83 days).”

The authors have anatomically defined cerebellar parcellations but have looked solely at total volume measures. What is the rationale for this? If there are differential impacts on cerebellar volume with age (Han et al., 2022; Bernard & Seidler, 2013), there may also be positive associations with behavior in regions that are less negatively impacted by volume. This would be consistent with the idea of reserve. One interesting set of correlations that could be considered is with respect to anterior lobules (I-IV and V) relative to the secondary motor representation in VIIIa and VIIIb, such that the latter may show a more robust association with behavior in the positive direction if volume in these regions is less impacted by aging.

As mentioned in response to one comment from the other reviewer, we investigated this question in our latest preprint (https://doi.org/10.64898/2026.02.13.705695). In this analysis, we did not find any relation between cerebellar outcomes and anatomical or functional cerebellar regions.

We consider this to be beyond the scope of the present paper, which focuses on the behavioral performances. The total cerebellar volume was added to show that the subject sample we used did actually exhibit atrophy in the cerebellum, but the purpose of the paper was not to focus on the link between structure and function.

With respect to timing, I recognize that the clock variance is insignificant based on p=.06. However, this is a relatively "close" result. I am very much of the mindset that things are significant or not. Inclusion of Bayesian analyses helps this, but I don't find this particularly convincing. The larger sample of individuals over age 80 is certainly a strength, and I'm not especially concerned about power. But I do wonder about overinterpretation. I would also emphasize the large degree of variability here in the oldest sample. This raises questions about associations with cerebellar metrics. This argument for relative preservation/reserve may be strengthened by looking at individual differences in structure relative to behavior. That is, in areas of the cerebellum where structure is less impacted by aging (as this is not entirely uniform) does this volume predict better behavior in this sample?

As noted earlier, the relationship between structure and function is examined in our other paper (https://doi.org/10.64898/2026.02.13.705695). Unfortunately, we were unable to include the 80+ group in that analysis because MRI data was available for only 20 older‑old participants and correlations/regression with 20 people are vastly underpowered.

We also want to point out that the almost significant difference highlighted by the reviewer between age groups actually goes in the direction of the older participants performing better than the young participants.

The note about the amount of variance in the older-old participants is fair, though.

The comparison with the Cam-CAN data set seems to be largely qualitative. Why did the authors not make a direct comparison to determine relative similarity in their sample compared to Cam-CAN? This would be a bit more compelling, though I suspect the differences are not statistically reliable (they note the oldest-old in the Leuven sample have a slightly larger volume). I do realize there are sample size differences, but a matched random sub-sample could also be created out of Cam-CAN. Why did they not compute the quadratic model in the Leuven sample as well?

A quadratic model was not considered very meaningful in the Leuven sample because age was not measured as a continuous variable but categorized into three discrete age groups (which provides more power to look at age-related differences). Our goal was not to determine whether absolute cerebellar volumes matched across datasets, for example, by creating comparable age groups in the Cam‑CAN dataset, but rather to assess whether the pattern of age‑related effects in our sample aligned with those seen in a larger dataset. In our opinion, the current approach sufficiently demonstrates that the age‑related trends we observe are consistent with those reported in Cam‑CAN.

The analysis of relative cerebellar gray and white matter is quite interesting. However, what about regional patterns to this? It would be particularly interesting to know if some regions are more or less impacted or preserved relative to the cortex. The data are seemingly available based on the processing approach (at least for gray matter). Was a similar analysis also computed in Cam-CAN? Replicating this in an independent sample would also be of interest.

We agree with the reviewer that this is indeed interesting for further analyses on this dataset. However, it falls beyond the scope of the present paper. Our preprint (https://doi.org/10.64898/2026.02.13.705695) looks at regional patterns for the cerebellum. Other papers have compared age-related decline in different cortical and subcortical regions as discussed on p.35 of our discussion:

“Given that the cerebellum exhibited a relatively less pronounced structural decline compared to other brain regions as shown here and in another previous study (Taki et al., 2011), it seems more plausible that the cerebellum might compensate for deficits caused by structural changes in other areas rather than vice-versa. Age-related gray and white matter degeneration is usually faster in frontotemporal regions and subcortical regions, including the hippocampus, amygdala and thalamus than in the cerebellum (Fjell et al., 2013; Giorgio et al., 2010; Neufeld et al., 2022). Although this does not directly indicate functional implications, it suggests that cortical regions are less likely to compensate for cerebellar loss when they exhibit more severe degeneration.”

The authors argue for cerebellar reserve and present compelling behavioral data in support of this with their many tasks. In instances where they look at largely cerebellar-mediated measures, they demonstrate that older adults and the >80 year old group show relatively intact behavior, even those in the group for total cerebellar gray matter volume (and white matter) is significantly smaller than in young adults. As noted, the behavioral data are very compelling, and as an individual who looks at aging populations in their research, seeing areas and domains of preservation is always interesting and useful. This pattern certainly may be consistent with cerebellar reserve. However, it would be more compelling if the authors also looked at these behaviors with respect to cerebellar volume. That is, there is still a great deal of variability in behavior in the older and >80 samples (though also in the young adults) that may still be associated with cerebellar volume. Poorer performance may be present in those with smaller volumes. This would also be somewhat consistent with the notion that these tasks are those that are derived from work in cerebellar degeneration samples. Associations between behavior and cerebellar measures would speak to this. If there are no associations with volume, this would be particularly interesting and compelling in the context of reserve. Alternatively, if there are differential impacts on cerebellar volume with age (Han et al., 2022; Bernard & Seidler, 2013), there may also be positive associations with behavior in regions that are less negatively impacted by volume. This would be consistent with the idea of reserve. One interesting set of correlations that could be considered is with respect to anterior lobules (I-IV and V) relative to the secondary motor representation in VIIIa and VIIIb, such that the latter may show a more robust association with behavior in the positive direction if volume in these regions is less impacted by aging. Not all individuals completed the scan (due to safety and comfort considerations), which would limit statistical power potentially, but this could be conducted in the subset of individuals that have both sets of data.

This point overlaps with the issues raised by the other reviewer in comments 1 and 2, which highlights the importance of this point. Yet, we decided to address this analysis in a separate paper. In the current manuscript, our primary focus was on the behavioral aspects, as these are already quite extensive on their own. In our subsequent work (https://doi.org/10.64898/2026.02.13.705695), we conducted an in-depth investigation into the relationship between cerebellar-specific measures and cerebellar structure across distinct cerebellar regions (including anatomical regions and functionally defined regions according to the atlas of Nettekoven et al., 2024). We found that aging does not affect the cerebellum uniformly, but that some anatomical regions exhibit stronger age effects. For the functionally defined regions the age effects were uniform though. There was no relation between behavioral cerebellar-specific measures and anatomical or functional cerebellar regions.

Some of the assertions the authors make in the discussion about the cerebellum have less pronounced structural decline relative to other brain regions would benefit from being tempered. They used relative measures here, and this is certainly interesting. But, how do other regions stack up? What would the hippocampus look like if such a measure were used? And as noted, does this pattern replicate in the CAM-CAN sample? Further, the authors cite Jernigan et al. (2001) in arguing that cerebellar changes are smaller than those in other brain regions, when in looking at their tables, in fact, the gray matter reductions of the cerebellum are comparable to those of the prefrontal cortex and second only to those of the hippocampus.

We agree with the reviewer that this is an interesting question but this question needs to be addressed in a separate paper. We also remove the citation to the Jernigan paper.
